# Anterior insular cortex and the perception of internalized stigma and its components: a scoping review.

**DOI:** 10.1192/j.eurpsy.2024.1291

**Published:** 2024-08-27

**Authors:** N. Lutova, E. Gerasimchuk, M. Khobeysh, M. Bocharova, O. Makarevich, M. Sorokin

**Affiliations:** ^1^V.M. Bekhterev National Medical Research Centre for Psychiatry and Neurology, St.Petersburg, Russian Federation; ^2^King’s College London, London, United Kingdom

## Abstract

**Introduction:**

Personality neuroscience employs a broad range of methods to identify the neurobiological mechanisms of complex psychological phenomena. The role of the insula is often associated with its involvement in emotion processing.

**Objectives:**

The study aims to identify the associations between neural activity in the anterior insula cortex (AInC) and self-stigma (or its components) in a scoping review.

**Methods:**

We searched in PubMed (MEDLINE), PsychINFO, EMBASE via the Ovid platform through September 21st, 2022. Included studies had to use fMRI to assess neurophysiological markers in AInC, and to include a measure of association between fMRI results and a measure of self-stigma and/or its components as assessed by a scale or questionnaire in participants aged 18-65 y.o. The PRISMA-ScR checklist was used.

**Results:**

After full-text screening 10 of 206 original researches were chosen for the final analysis (Table 1).

**Table 1:** Included studies in the analysis.

1	DeWall et al. Soc Cogn Affect Neurosci. 2012; 7(2): 184-192.
2	Masten et al. Neuroimage. 2011; 55(1): 381-388.
3	Kross et al. Proceedings of the National Academy of Sciences. 2011; 108(15): 6270-6275.
4	Bolling et al. Neuroimage. 2011; 54(3): 2462-2471.
5	Lindner et al. PLoS One. 2014; 9(1): e85014.
6	Achterberg et al. Soc Cogn Affect Neurosci. 2016; 11(5): 712-720.
7	Muscatell et al. Brain Behav Immun. 2016; 57: 21-29.
8	Sankar et al. Front Behav Neurosci. 2019;13.
9	Cáceda et al. Clin Neurosci. 2020; 270(5): 619-631.
10	Landa et al. J Psychosom Res. 2020; 128: 109881.

In 5 studies, the results were presented with MNI-space coordinates. Figure 1 illustrats the regions of local activity change maxima according to MNI-space coordinates based on the results of the included studies in the analysis.

Neural activation in the regions of the AInC was positively associated with greater levels of social rejection sensitivity and other components of self-stigma in 9 studies. Reduced activity was observed in only one study (Lindner et al., PLoS One. 2014; 9(1): e85014) among highly self-stigmatized patients with schizophrenia. This finding may reflect a biological manifestation of deficits in self-awareness and affective processing in schizophrenia.

**Image:**

**Figure:**
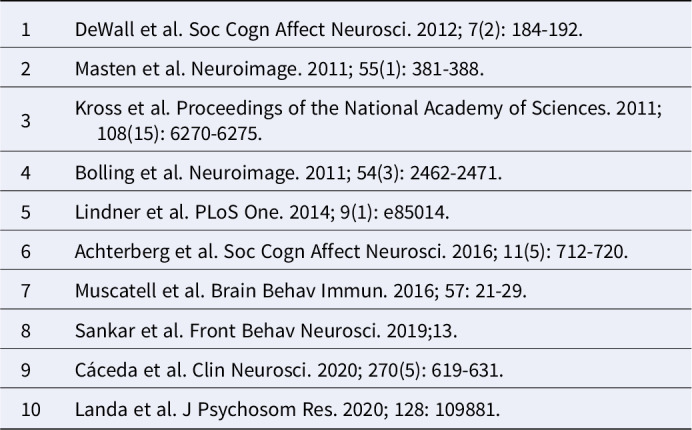

**Conclusions:**

Associations between neural activity changes in specific brain regions and levels of self-stigma and/or its components, as reported in included neuroimaging studies, have the potential to shed light on the neurobiological mechanisms underlying such a complex psychological phenomenon as stigma.

**Disclosure of Interest:**

None Declared

